# Dissecting the Genetic Complexity of Fusarium Crown Rot Resistance in Wheat

**DOI:** 10.1038/s41598-020-60190-4

**Published:** 2020-02-21

**Authors:** Shree R. Pariyar, Gul Erginbas-Orakci, Said Dadshani, Oyiga Benedict Chijioke, Jens Léon, Abdelfattah A. Dababat, Florian M. W. Grundler

**Affiliations:** 1 Present Address: Forschungszentrum Jülich GmbH, Institut für Bio- und Geowissenschaften (IBG)-2, Pflanzenwissenschaften, D-52425 Jülich, Germany; 2 Institute of Crop Science and Resource Conservation (INRES), Molecular Phytomedicine, Karlrobert- Kreiten Strasse 13, D-53115 Bonn, Germany; 3 International Maize and Wheat Improvement Centre (CIMMYT), P.K. 39 06511, Emek Ankara, Turkey; 4 Institute of Crop Science and Resource Conservation (INRES), Plant Breeding, Katzenburgweg 5, D-53115 Bonn, Germany

**Keywords:** Plant breeding, Plant immunity

## Abstract

Fusarium crown rot (FCR) is one of the most important diseases of wheat (*Triticum aestivum* L.). FCR is mainly caused by the fungal pathogens *Fusarium culmorum* and *F. pseudograminearum*. In order to identify new sources of resistance to FCR and to dissect the complexity of FCR resistance, a panel of 161 wheat accessions was phenotyped under growth room (GR) and greenhouse conditions (GH). Analysis of variance showed significant differences in crown rot development among wheat accessions and high heritability of genotype-environment interactions for GR (0.96) and GH (0.91). Mixed linear model analysis revealed seven novel quantitative trait loci (QTLs) linked to *F. culmorum* on chromosomes 2AL, 3AS, 4BS, 5BS, 5DS, 5DL and 6DS for GR and eight QTLs on chromosomes on 3AS, 3BS, 3DL, 4BS (2), 5BS, 6BS and 6BL for GH. Total phenotypic variances (*R²*) explained by the QTLs linked to GR and GH were 48% and 59%, respectively. In addition, five favorable epistasis interactions among the QTLs were detected for both GR and GH with and without main effects. Epistatic interaction contributed additional variation up to 21% under GR and 7% under GH indicating strong effects of environment on the expression of QTLs. Our results revealed FCR resistance responses in wheat to be complex and controlled by multiple QTLs.

## Introduction

Bread wheat (*Triticum aestivum* L) is an allohexaploid derived from a combination of three closely-related but independently maintained genomes (A, B and D) formed by multiple hybridization events among the three different progenitor species. The first hybridization occurred between the wild diploid wheat *T. urartu* (AA, 2n = 14) as the A-genome donor and an unknown species containing the B genome (BB, 2n = 14, most probably *Aegilops speltoides*), resulting in the tetraploid ancestor of modern *Triticum* species, wild emmer wheat *T. turgidum* ssp. *dicoccoides*, AABB, 2n = 28), which further hybridized with goat grass *A. tauschii* (DD, 2n = 14) to form modern bread wheat^1–4^. Wheat is the major food crop consumed in the world which contains the main source of calories (19%) and proteins (20%) in human diets and contributes significantly to animal feeds^5^. Fusarium crown rot (FCR) is one of the most prevalent and devastating diseases of wheat and barley^6,7^. Affected plants suffer from necrosis and dry rot of the crown, basal stem and root tissue and may finally die at a premature stage. FCR is caused mainly by the fungi *Fusarium culmorum* and *F. pseudograminearum*. Both species produce toxic secondary metabolites (mycotoxins) that pose a serious health risk to humans and livestock^8^.

In the past decade, numerous quantitative trait loci (QTLs) linked to FCR resistance have been reported. However, the degree of resistance contributed by individual QTLs is relatively small and often based on multiple genes^9–12^. Moreover, many QTLs were linked to FCR resistance at different stages of plant growth, e.g. seedling stage or adult plant resistance^13^. The most significant and durable resistance QTL was identified on chromosome 4D for Fusarium head blight (FHB) caused by *F. culmorum*^14^. Another major QTL, *Fhb1*, conferring resistance to FHB caused by the closely related pathogen *F. graminea*rum, was found on chromosome 3BS (Qfhs.ndsu) in spring wheat^15–18^. Recently, Erginbas-Orakci *et al*.^19^ reported two QTLs on chromosomes 3BS linked to FCR resistance in a CIMMYT spring wheat mapping panel under green house conditions^19^. However, since breeding FCR resistance into wheat is a complex process, so far no durable FCR resistant wheat varieties are available. This is mainly due to the nature of disease development, pathogen species-specific host response, nature of inheritance, and strong genotype-by-environment interaction^20–23^. Further, molecular mechanisms of FCR resistance and interaction with environment are poorly understood. Thus, characterizing potential epistatic interactions between QTLs and environment will provide new insight in understanding the genetic basis of FCR resistance.

Wheat accessions of different origin comprise a broad genetic diversity which can be exploited to breed for high yielding and disease resistant cultivars. Current progress in high density mapping, sequence availability and statistical tools make it possible to uncover the majority of QTL effects and functionally characterize their role in host-pathogen interactions. Genome-wide association study (GWAS) is a powerful approach to detect associations between phenotypic variation and genetic polymorphisms; in this way, QTLs for traits such as FCR can be located in the genome. GWAS utilizes linkage disequilibrium to dissect the genetic architecture of complex traits by correlating phenotypes to genotypes. It has been used earlier to identify QTLs that are involved in plant responses to soil borne pathogens in a large set of unrelated wheat accessions^19,24^. Detection of QTLs tightly linked to gene/s that control FCR resistance are the pre-requisite for marker assisted breeding. In the present study, we analyze the genetic basis of the FCR infection, disease development, and the interaction accross multiple environments. Our study not only confirmed previously described QTLs linked to FCR but also identified novel QTLs that have additive main and epistatic effects on FCR resistance which upon functional validation would provide additional surrogate markers for screening FCR in wheat.

## Results

### Diverse wheat accessions responded differentially to *F. culmorum* crown rot

**Figure 1:**
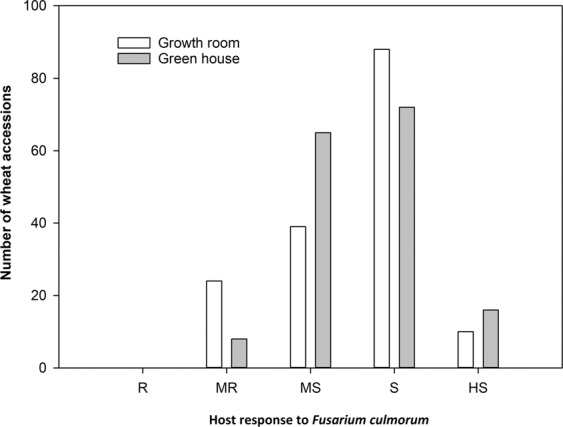
Response of 161 diverse wheat accessions to *Fusarium culmorum* under growth room and greenhouse trials (R resistant, MR moderately resistant, MS moderately susceptible, S susceptible, and HS highly susceptible).

Screening of 161 wheat accessions revealed high variability in host response to *F. culmorum* infection and disease development. Analysis of variance showed significant variation in FCR among the wheat accessions under GR and GH. Additionally, FCR disease scores were found to be highly heritable (Table 1) ranging from 91.0% to 96.0%. The results revealed 15% of the 161 wheat accessions to be moderately resistant, 24% moderately susceptible, 55% susceptible and 6% highly susceptible to *F. culmorum* under GR; whereas 5% of the 161 wheat accessions were moderately resistant, 40% moderately susceptible, 45% susceptible, and 10% highly susceptible to *F. culmorum* crown rot under GH (Fig. 1). Correlation between the two individually repeated experiments was highly significant for both GR (*R²* = 0.78) and GH (*R²* = 0.66). Twenty four wheat accessions showed immune responses to FCR under GR and eight accessions under GH (Table S1).

**Table 1:** Analysis of variance for *Fusarium culmorum* disease development in 161 diverse wheat accessions across growth room and greenhouse.

Trait	LSmean	S.V.	D.F	MS	Sig.	Heritability (%)
FCR under growth room	2.92	accessions	160	6.29	***	96.0
		Error	1449	0.28		
FCR under greenhouse	2.90	accessions	160	2.51	***	91.0
		Error	1440	0.21		

S.V. source of variance, D.F degree of freedom, MS sum of mean squares, Sig. significance level at ***P < 0.001.

### Density of polymorphic SNPs differs among wheat sub-genomes A, B and D

**Figure 2:**
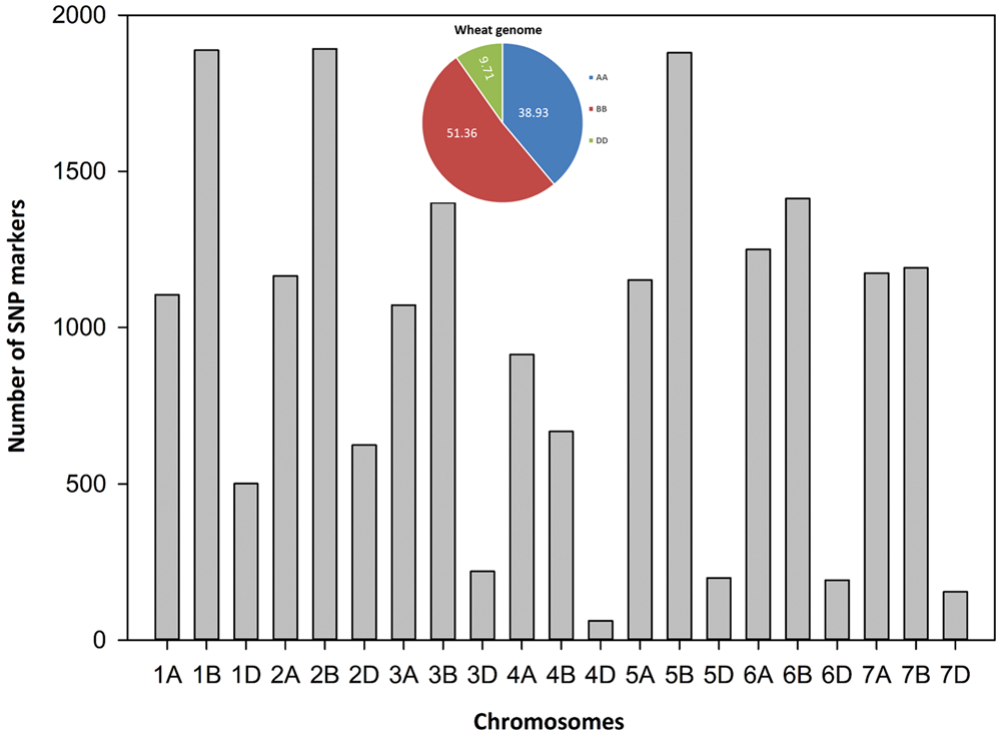
Distribution of single nucleotide polymorphism (SNP) markers across wheat genome.

After the exclusion of all monomorphic markers (MAF < 5% and missing data>5%), a total of 23,364 polymorphic SNPs was obtained. Among them, 86% (20,116 SNPs) were found to be mapped in the 90K SNP consensus map. The results showed an uneven distribution of polymorphic SNP markers across wheat genomes A, B, and D (Fig. 2). The highest number of SNPs was mapped on B (10,331, 51.36%) and A (7831, 38.93%) genome, whereas a very low number of polymorphic SNPs (1954, 9.85%) was located on D genome. The total genomic distance covered by total SNP markers in 161 wheat accessions was 3653 cM. The highest number of SNPs were located on chromosome 2B (1892 SNPs, 9.71%), while the lowest number found on chromosome 4D (62 SNPs, 0.31%). On average, a single marker was mapped on 18.15 cM distance across 21 wheat chromosomes.

### LD differs aong 21 chromosomes in 161 diverse wheat accessions

**Figure 3:**
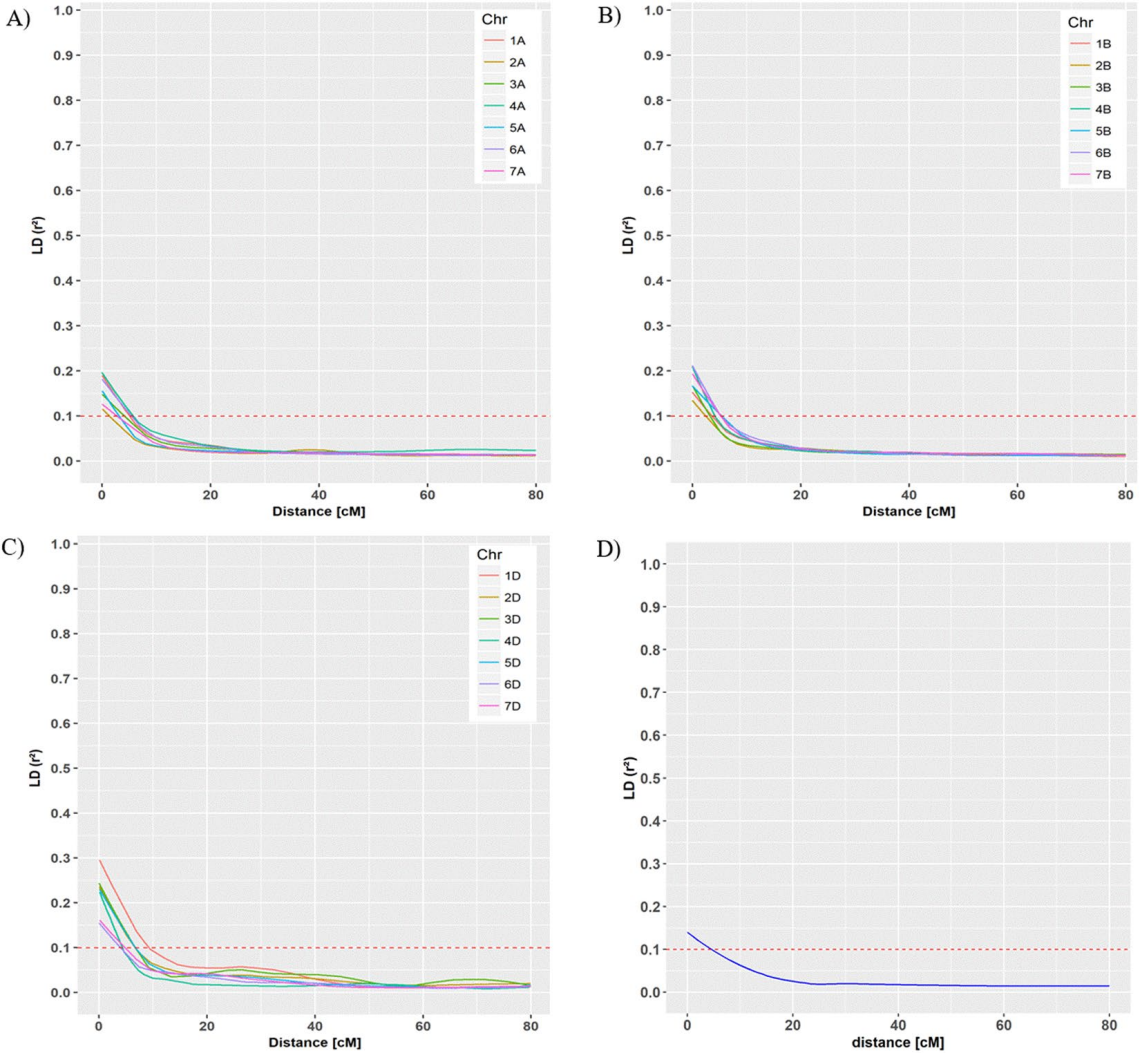
. Genome-wide linkage disequilibrium (LD) decay across wheat genomes A, B and D. The *r*^2^ values of LD were plotted against the genetic distance in centiMorgan (cM) across all chromosomes. (**A**) LD across A genome, (**B**) LD across B genome, (**C**) LD across D genome, and (**D**) LD across whole ABD genome. LD value is considered below *r*^*2*^ = 0.1 threshold. The horizontal red dotted line marks the threshold above which LD is likely due to genetic linkage.

A total of 20,116 mapped SNPs were used to estimate genome-wide LD. The *r*^2^ values of LD were plotted against the genetic distance in cM. The critical value for LD was 0.1, and above this value LD was considered to be due to genetic linkage. The genome wide LD across A, B and D genomes were 3.9, 4.19 and 6.06 cM, respectively (Fig. 3). The genomic region of 3.9 to 6.06 cM to each side of the significant marker-trait associations on chromosomal arm were defined as a “QTL-region”. All the significant associations in these regions were designated as single QTL. LD decay varied among chromosomal regions across wheat sub-genomes (A, B, or D). The highest LD value was recorded on chromosome 2A, while lowest LD was found on chromosome 1D.

### PCA analysis revealed minimum genetic sub-structure among 161 diverse wheat accessions

**Figure 4:**
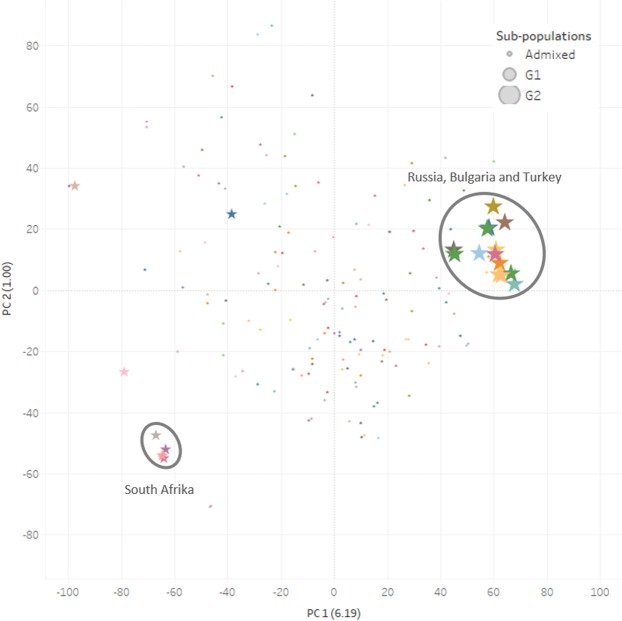
Principal components analysis separated 161 diverse wheat accessions into two groups. A scatter plot of principal component 1 (PC1) plotted against principal component 2 (PC2). Each symbol represents a wheat accession in mapping panel. G1 represents group 1 while G2 represents group 2.

PCA analysis estimated the mapping wheat panel into two sub-groups (Fig. 4). The first three principal components explained 8.54% of the total variation. The low genetic variation exhibited by first PC (6.19) did not show a distinct sub-structure even though the studied panel includes landraces, breeding lines and cultivars. The first group consisted of 89 winter wheat accessions mostly originating from South Africa (5), and Iran (2), including the United States (1), whereas the second group was composed of 72 wheat accessions mainly originated from Russia (8), Bulgaria (2), Moldova (2), including Turkey-CIMMYT ICARDA (3) (Table S1).

### GWAS identified novel and confirmed previously described QTLs linked to *F. culmorum* CR at multiple environment

**Figure 5:**
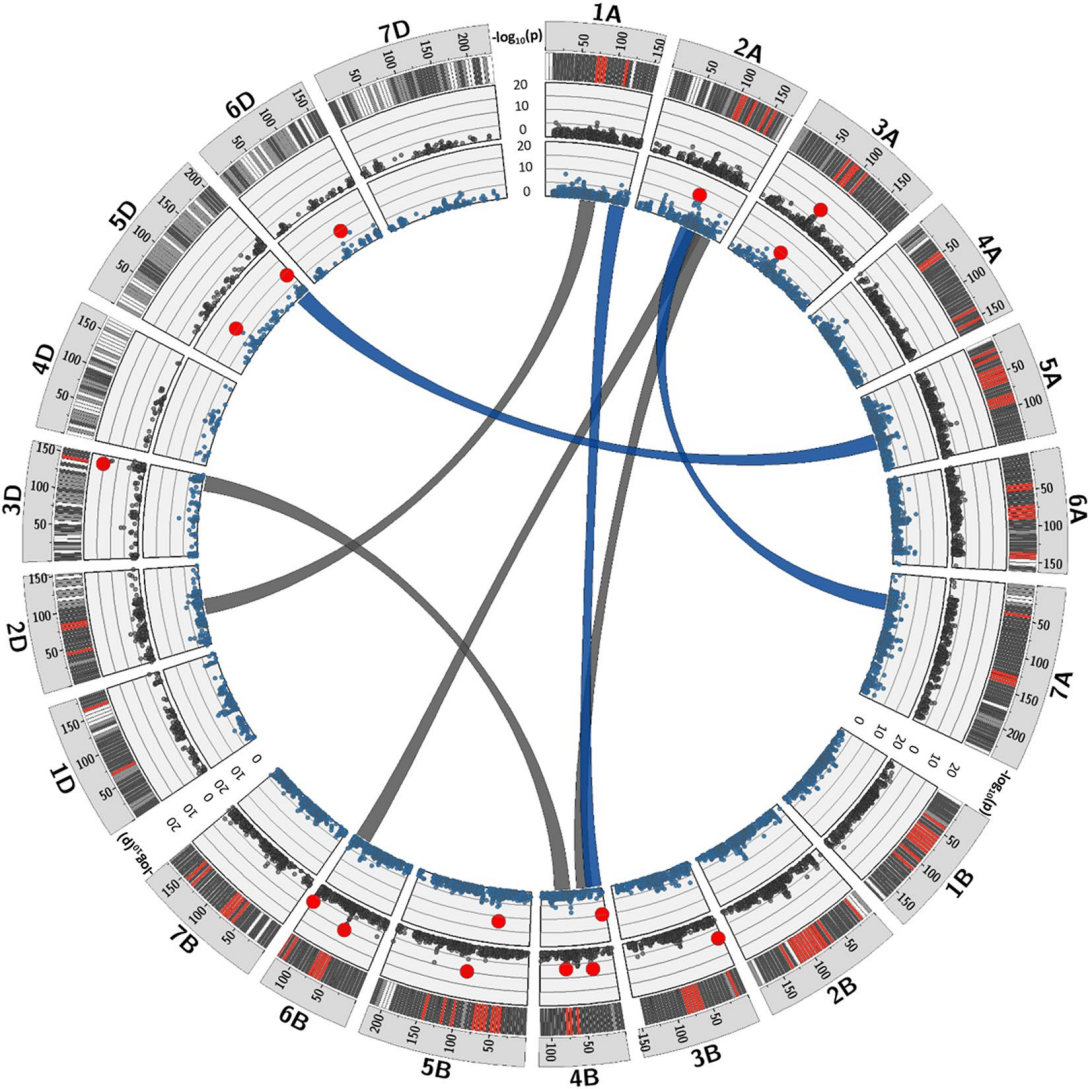
Genetic architecture of *Fusarium* crown rot (FCR) resistant in 161 diverse wheat accessions. Outer circle represents distribution of SNPs on wheat chromosomes from white (low density) to red (high density). Two inner blue and black circles represent the Manhattan plots for corresponding QTLs (bold red) linked to FCR under growth room and greenhouse environment respectively. The epistatic interaction between the QTLs are indicated by blue (GR) and black (GH) connector links in the center of the plot.

QTLs associated with FCR resistance were identified individually for each environment by using a mixed linear model with kinship matrix (MLM-P + *K*). Seven QTLs on chromosomes 2AL, 3AS, 4BS, 5BS, 5DS, 5DL and 6DS were linked to FCR resistance under GR, whereas 8 QTLs on chromosomes 3AS, 3BS, 3DL, 4BS, 5BS, 5BL, 6BS, and 6BL were linked to FCR resistance under GH (Fig. 5). QTLs above LOD value ≥ 6 were considered significant. The QTLs were detected on all three sub-genomes even though distribution of the polymorphic SNPs on D genome was comparatively low. The highest number of QTLs (3) were identified on chromosome 4BS followed by chromosomes 3AS and 5BS with two QTLs each. The alleles linked to these QTLs showed moderate effect (*R*^2^ = 5.80 to 11.22%) to reduce FCR infection and disease development (Table 2). Under GR, the strongest effect was recorded by the QTL on chromosome 5BS (*R*^2^ = 9%), while under GH, the strongest effects was exhibited by the QTLs on chromosomes 5BL and 3DL accounting 12.27 and 11.22% GH, respectively (Table 2). Collectively, all QTLs identified for GR and GH explained 48% and 59.48% of the observed variation (Table 2). The genomic regions on chromosomes 3AS, 4BS and 5BS were linked to FCR resistance in both GR and GH trials and showed similar QTL effects in both environments. Since this region showed a stable response towards FCR at both environmental conditions, these QTLs might be promising candidates for breeding FCR resistance. The genomic regions linked to two QTLs on chromosomes 4BS and 6DS under GR and three QTLs on chromosomes 3BS, 4BS and 6BS under GH were previously identified as resistance QTLs for FHB disease incidence, spread and severity^17,25–27^.

**Table 2:** QTLs linked to *Fusarium culmorum* crown rot under growth room and greenhouse.

a) growth room												
SN	Marker	CHR	POS (cM)	MAF	Genetic region	P-LOD	P-FDR	Probability	Allele	Effect (*R²*)	Heritability (*H²*)	SE of *H²*
1	Kukri_c57491_156	2AL	104,13	0,47	104,13	12,33	4,60172E-09	4,70283E-13	T/C	6,93	0,96	*P* < *0.001*
2	wsnp_Ra_c16278_24893033	3AS	83,85	0,36	83.31–83.85	9,70	1,95925E-07	2,0023E-10	A/G	5,87		
3	wsnp_Ku_c12399_20037334	4BS	6,78	0,30	6,77	9,61	2,20756E-07	2,48167E-10	T/G	7,07		
4	wsnp_Ku_c17875_27051169	5BS	55,52	0,43	55.52–55.52	11,64	1,11631E-08	2,28168E-12	T/C	9,11		
5	RAC875_rep_c111521_246	5DS	67,49	0,06	67.49–67.50	10,53	7,22785E-08	2,95466E-11	T/G	5,47		
6	Excalibur_c2795_1518	5DL	198,19	0,41	198,18	9,01	6,38067E-07	9,78131E-10	T/C	7,52		
7	BS00021881_51	6DS	98,11	0,45	98,10	9,45	2,86754E-07	3,51665E-10	A/G	6,13		
Sum									48,09			
**b) greenhouse**												
1	CAP8_c1393_327	3AS	90,55	0,44	90,55	9,39	5,57516E-07	4,08865E-10	A/G	7,19	0,91	*P* < *0.001*
2	CAP12_rep_c3868_270	3BS	5,86	0,28	5,85	8,35	4,71367E-06	4,44453E-09	T/G	7,82		
3	wsnp_Ex_c14027_21925404	3DL	143,01	0,23	143,01	15,42	3,64073E-12	3,81428E-16	T/C	11,22		
4	tplb0045c06_1675	4BS	34,15	0,06	34,14	7,24	3,2349E-05	5,76147E-08	T/C	6,05		
5	RAC875_rep_c72961_977	4BS	75,65	0,20	75,64	6,56	0,000104516	2,73745E-07	T/C	6,24		
6	Excalibur_c23304_353	5BS	95,43	0,20	95.43–95.44	9,81	2,487E-07	1,56333E-10	T/G	9,44		
7	RAC875_c17297_341	6BS	58,20	0,27	58.19–58.20	10,06	1,66294E-07	8,71105E-11	T/C	5,80		
8	BobWhite_c19298_97	6BL	122,26	0,21	122,25	6,75	7,64241E-05	1,76148E-07	T/C	5,73		
Sum									59,48			

*CHR chromosomes, POS position, cM centi Morgan, MAF marker allele frequency, FDR false discovery rate, *R²* explained genetic variation, SE standard error, markers in bold reprent the QTLs located on same chromosomes.

### Epistatic interaction affects the expression of *F. culmorum* infection and disease development

Epistatic QTL interactions (with and without the main effects) were detected between each 10 pairs of QTLs for FCR resistance under GR and GH (Fig. 5). Those digenic interactions between the QTLs under GR account for 12.24–16.14% of the phenotypic variation, whereas the interactions detected under GH explained between 9.64–20.11% of the phenotypic variation (Table 3). The majority of those epistatic interactions involved no main effect QTL. However, an epistatic interaction involving main effect QTL on chromosome 4BS (6.78 cM) * QTL on chromosome 2AL (104.13 cM) was detected under GR which explained added 5.2% phenotypic variation. Interestingly, an interaction between two main effect QTLs (QTLs on chromosomes 4BS (75.65 cM) * 3DL (143.01 cM) for GH trial with additional 2.65% variation were observed. These results suggest that multiple QTLs (additive-additive epistatic interactions) can influence the expression of FCR resistance.

**Table 3:** Epistasis interaction linked to *Fusarium culmorum* crown rot under growth room and greenhouse.

a) growth room											
SN	Marker_1 (M1)	CHR	POS (cM)	Marker_2 (M2)	CHR	POS (cM)	P-LOD	P-FDR	Prob_F	Allele (M1*M2)	Effect (*R²*)
1	Ex_c3201_1046	1AL	139,74	wsnp_Ku_c12399_20037334	4BS	6,78	19,78	8,00863E-20	1,65228E-20	(T/C)*(T/C)	12,24
2	BS00022896_51	2 AS	109,52	wsnp_Ra_c63822_63288359	7AS	56,49	19,78	2,95702E-33	1,65228E-20	(T/C)*(T/C)	15,75
3	Excalibur_c2795_1518	5DL	198,19	Tdurum_contig55097_601	5AS	89,56	17,77	4,91127E-18	1,70502E-18	(T/C)*(T/C)	12,95
4	BS00021881_51	6DS	98,11	BS00040933_51	5AS	36,73	35,72	1,11928E-22	1,90877E-36	(A/G)*(A/G)	16,14
5	RFL_Contig2200_1024	7AL	120,68	Tdurum_contig43252_1762	3BS	37,29	23,05	6,88739E-16	8,89671E-24	(T/C)*(T/G)	12,01
	Sum										69,09
**b) greenhouse**											
1	Excalibur_c7282_285	2AL	122,54	Kukri_c45876_157	6BL	120,61	14,51	2,28164E-14	3,1055E-15	(T/C)*(T/C)	9,64
2	Kukri_c22553_60	2DS	73,20	Tdurum_contig32437_257	1AS	81,51	15,98	1,35877E-15	1,04787E-16	(T/C)*(A/G)	13,34
3	Kukri_c40909_784	3BS	65,72	wsnp_Ex_c6400_11123059	2DS	38,25	12,90	5,23841E-13	1,26144E-13	(T/C)*(A/G)	11,70
4	Excalibur_c18318_701	4BS	32,66	Excalibur_c7241_284	2AL	143,22	17,33	9,65719E-17	4,65109E-18	(A/G)*(A/G)	11,62
5	RAC875_rep_c72961_977	4BS	75,65	wsnp_Ex_c14027_21925404	3DL	143,01	32,41	1,52315E-29	3,88956E-33	(T/C)*(T/C)	20,11
	Sum										66,42

*CHR chromosomes, POS position, cM centi Morgan, F-value epistatis fischer test value, Prob probability, FDR false discovery rate, *R²* explained genetic variation, all epistatic interactions were significant at *P* < *0.001*, markers in bold represent QTLs with main effects.

### *In-silico* analysis predicts defense related genes linked to *F. culmorum* CR

*In-silico* analysis predicted the majority of the identified genes/proteins to be involved in diverse biological processes such as carbohydrate transport and metabolism, signal transduction, cell cycle control, and response to stimuli, respectively. For instance, eleven of the identified QTLs on chromosomes 2AL, 3AS, 5DS, 5DL, 3BS, 3DL, 4BS, 6BS and 6BL were linked to genes involved in plant-pathogen interactions (Table 4). One QTL on chromosome 5BS was found to linked to a gene predicted to be involved in abiotic stress. Three other QTLs on chromosomes 4BS, 3AS, and 5BS were linked to genes/proteins with unknown function. The details of *in-silico* identification of intra-chromosomal locations of SNPs, and co-localized genes, allelic change, and their putative function is listed on Table 4. Annotation of the flanking sequences of marker CAP12_rep_c3868_270 (5.86 cM) at 3BS identified the Traes_3B_1396EA938.2 (W5CNP7) gene that harbours NAD(P)(+)-binding protein linked to *Fhb1* (Table 4). NAD(P)(+)-binding proteins were reported previously to enhance FHB resistance in wheat^28^. Similiarly, genes/proteins linked to the QTLs on 4BS, 6DS (Traes_6DL_E31AB6EED (W5H0C4)/Traes_6DL_5BE701A64 (W5GWI8)), 6BS Traes_6AS_33690B236 (W5GCQ4) and 6BL (Traes_6BL_9CFA54D4A, W5GLE7) were reported to reduce FHB spread and severity. Annotation of epitasis QTL on chromosomes 5AS, 2AL and 4BS detected proteins that are linked linked to FHB spread, severity and deoxynivalenol production. Moreover, we also found two other proteins associated with epi-QTLs on chromosomes 5AS and 7AL that were previously reported to retain *Fhb1* linked to FHB infection and severity^29^.

**Table 4:** *In-silico* identification of genes/proteins underlying QTLs linked to *Fusarium* crown rot and their putative functions under growth room and greenhouse.

a) growthroom										
SN	QTL	CHR	Allele	Blast annoation	Acce. no.	Genes/proteins	Amino acid change	Types of change (I)	Types of change (II)	Putative functions
1	Kukri_c57491_156	2AL	T/C	Triticum aestivum genome assembly, chromosome: 2A	LS992083.1	Traes_2AL_3F9271302 (W5ARG8)	L- > L	Transition	synonymous	Lipid transport and metabolism, and signal transduction mechanisms
2	wsnp_Ra_c16278_24893033	3AS	A/G	Triticum aestivum mRNA, clone: tplb0013i15, cv. chinese spring	AK456680.1	Traes_3AS_EBD285F1A (W5CNP7)	S- > S	Transition	synonymous	Cell cycle control, translation, posttranslational modification, protein turnover, and transcription
3	wsnp_Ku_c12399_20037334	4BS	T/G	Triticum aestivum genome assembly, chromosome: 4B	LS992090.1	Putative uncharacterized protein	T- > A	Transition	nonsynonymous	.
4	wsnp_Ku_c17875_27051169	5BS	T/C	Triticum aestivum genome assembly, chromosome: 5B	LS992093.1	Traes_5BL_BE78D7104 (W5FHE1)	T- > T	Transition	synonymous	Response to stress
5	RAC875_rep_c111521_246	5DS	T/G	Triticum aestivum genome assembly, chromosome: 5D	LS992094.1	Traes_5DS_ED246C623 (W5G4N2)	no_hit	Transversion	.	Carbohydrate transport and metabolism
6	Excalibur_c2795_1518	5DL	T/C	Triticum aestivum genome assembly, chromosome: 5D	LS992094.1	Traes_5DL_DB93C2CFF (W5FZH6)	no_hit	Transition	.	Protein phosphatase type 2A regulator activity and signal transduction
7	BS00021881_51	6DS	A/G	Triticum aestivum cultivar Chara chromosome: 6D alpha-amylase 1 gene, complete cds	KY368733.1	Traes_6DL_E31AB6EED (W5H0C4)/Traes_6DL_5BE701A64 (W5GWI8)	M- > T	Transition	nonsynonymous	Alpha-amylase activity; calcium ion binding; carbohydrate metabolism
**b) greenhouse**										
1	CAP8_c1393_327	3AS	A/G	Triticum aestivum genome assembly, chromosome: 3A	LS992086.1	Putative uncharacterized protein	no_hit	Transition	.	.
2	CAP12_rep_c3868_270	3BS	T/G	Triticum aestivum genome assembly, chromosome: 3B	LS992087.1	Traes_3B_1396EA938.2	no_hit	Transversion	.	NADPH binding protein, 1-deoxy-D-xylulose-5-phosphate reductoisomerase activity; isoprenoid biosynthetic process; metal ion binding;
3	wsnp_Ex_c14027_21925404	3DL	T/C	*Triticum aestivum* genome assembly, chromosome: 3D	LS992088.1	Traes_3DL_0421BBCE9 (W5D7G9)	N- > N	Transition	synonymous	Cell wall/membrane/envelope biogenesis, carbohydrate transport and metabolism
4	tplb0045c06_1675	4BS	T/C	Triticum aestivum genome assembly, chromosome: 4B	LS992090.1	Traes_4BS_4E68C8E47 (W5EAP7)	N- > N	Transition	synonymous	Signal transduction mechanisms, carbohydrate transport and metabolism, translation, ribosomal structure and biogenesis
5	RAC875_rep_c72961_977	4BS	T/C	Triticum aestivum genome assembly, chromosome: 4B	LS992090.1	Traes_4BL_1A04707C5 (W5E1P8)	I- > V	Transition	nonsynonymous	Transcription, translation, ribosomal structure and biogenesis, and posttranslational modification
6	Excalibur_c23304_353	5BS	T/G	Triticum aestivum genome assembly, chromosome: 5B	LS992093.1	Putative uncharacterized protein	no_hit	Transversion	.	.
7	RAC875_c17297_341	6BS	T/C	Triticum aestivum genome assembly, chromosome: 6 A	LS992095.1	Traes_6AS_33690B236 (W5GCQ4)	Q- > Q	Transition	synonymous	Amino acid transport and metabolism, mannan synthase activity, nucleotide-diphospho-sugar transferases
8	BobWhite_c19298_97	6BL	T/C	Triticum aestivum genome assembly, chromosome: 6B	LS992096.1	Traes_6BL_9CFA54D4A (W5GLE7)	A- > A	Transition	synonymous	Transcription, translation, ribosomal structure and biogenesis, and posttranslational modification

*SN serial numbers, QTL quantitative trait loci, CHR chromosomes, A adenine, T thymine, G guanine, C cytosine, Acce. no accession number, L leucine, S serine, T threonine, A alanine, M methionine, N asparagine, Q glutamine, NADPH nicotinamide adenine dinucleotide phosphate, annotations were considered positive when the database sequence was lower than e-value0.0e-15.

## Discussion

A major global priority of plant breeding and plant pathology is to develop high yielding disease resistant wheat varieties to specific biotic and abiotic stresses. FCR is a major biotic factors reducing wheat yield especially in drought persistent regions of the world. Despite the general awareness of damage caused by *F. culmorum*, no durable resistant cultivar has been released so far. Lack of sufficient knowledge, technical know-how and limited understanding of the genetic basis have been the key challenges in developing resistant cultivars. In fact, developing FCR resistant cultivars is difficult, due to the technical challenges associated with CR screening as well as complex genetic background consisting of multiple genes/QTLs with small or intermediate effects^30,31^. Therefore, understanding the complexity of FCR infection and disease development, and their interaction with environment will be crucial for further progress.

Various screening approaches have previously been tested and colonized grain method was identified as the most reliable method^19^. Standard wheat “check lines” with known resistance/susceptibility to *F. culmorum* were included into the trials to evaluate the response of the diverse wheat populations. The results demonstrated that diverse wheat accession contribute a board range of genetic variation towards FCR infection and disease development under GR and GH. FCR disease development is highly correlated between two successive trials. High heritability (Table 1) found in this study indicated that the observed phenotypic variation is mostly due to genetic factors, and suggested a potential transmission of alleles to successive generations. We found FCR infection and development varied significantly by genotype x environment, and suggested that FCR development differ under different environmental conditions. For example, more numbers of resistant accessions were recorded under GR compared to GH, as GR has minimal environmental effect. Similar to our finding, Erginbas-Orakci *et al*.^19^, confirmed that FCR disease development is comparatively higher in GR compared to GH and field^19^. Mitter *et al*.^32^, reported a significant role of environment on FCR disease development at multiple environment^32^.

Genotyping with 90K SNP marker showed that the distribution of polymorphic SNPs differs among A, B, and D genomes in the wheat panel indicating that rate of recombination varies among sub-genomes. The process of effective recombination events in A and B genomes might have resulted from the historical development of hexaploid wheat and its domestication process^2,33^. Consistent with our previous findings, A and B genomes have higher polymorphism than D genome^19,24^. Würschum *et al*.^34^ reported similar finding of few SNPs to be located on the D genome of 172 European winter wheat cultivars^34^. The number of mapped markers in the D genome is usually 3 to 5 fold lower compared to A and B genomes^35,36^. In fact, the low genetic diversity in D genome is very common in wheat. However, our study represents the highest number of polymorphic SNPs (20116) available in current wheat physical maps^37^, (updated in 2016).

PCA analysis detected a low genetic sub-structure in the studied wheat mapping panel. No specific sub-groups were found in the mapping panel, which might reflect the fact that CIMMYT has exchanged germplasm within its breeding program worldwide. This is important, since population structure and relatedness among individuals can cause false associations between markers and traits^38,39^. To adress false signals, we applied multiple linear models (P+K), taking into consideration of principle component analysis (P) and relatedness (K). The corrected models separate the true functional signal from false positives. In fact, genetic variability within the studied mapping panel turned out to be very low so that no further correction was required. Similar to our finding, in other studies with diversified wheat and *A. tauschii* populations population structure was found to vary according to mapping population resulting in two sub-groups^40,41^.

The principle of GWAS analysis is based on LD. The LD decay over genetic distance in a mapping population helps to measure the density of the marker required to cover the genomic distance while; a rapid LD decay suggests the number of markers required for association analysis^42^. Our results showed that LD decay differs among wheat sub-genomes (Fig. 3). Similar to our findings, Neumann *et al*.^40^ has reported the inconsistency of LD across the whole genome, sub-genomes as well individual chromosomes^40^. Estimated LD decay in this study supports the LD in self-pollinated plants which is comparatively smaller (4 cM) than previously reported 5 cM for wheat (Fig. 3D)^43^. Selection of favorable phenotypes by International Winter Wheat Improvement Program (IWWIP) during breeding history might have created a smaller LD in the studied mapping panel. The other reason could be the use of the high density SNP marker genotyping platform (90K SNPs). High density markers helps to capture the genomic regions influenced by a short and intense breeding history. In consistence with our study, LD decay of <5 cM in 157 wheat landraces and 189 Canadian bread wheat accession, and 5–10 cM for 93 Chinese bread wheat lines were previously reported^44,45^. However, LD depends on many factors such as process of domestication, population sub-division, founding events and selection, and can therefore differ from population to population^46^.

**Figure 6:**
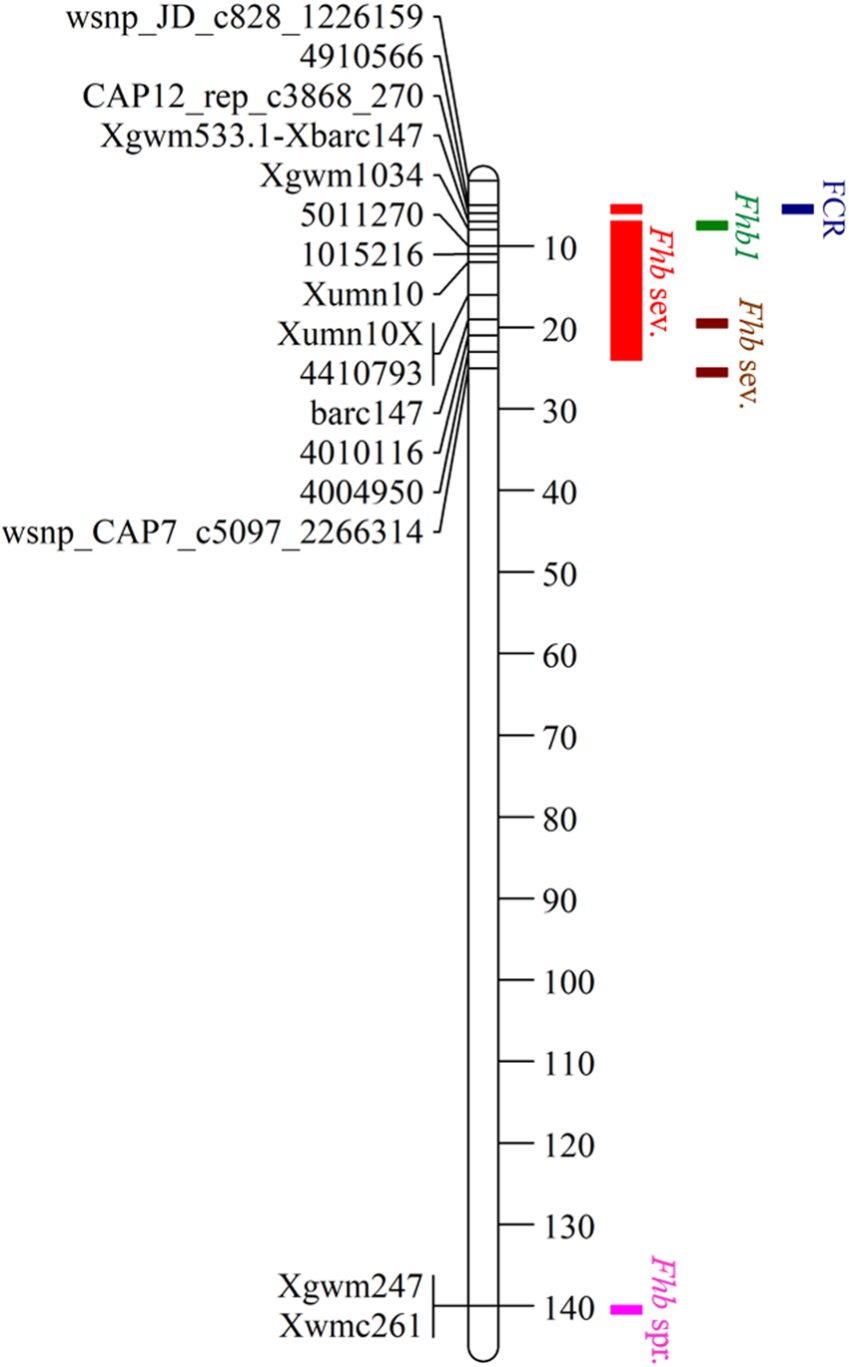
Distribution of major QTLs linked to *Fusarium* head blight (*Fhb*) and *Fusarium* crown rot (FCR) disease resistance on wheat chromosome 3B, *Fhb sev.=*Fhb* severity, *Fhb* spr. =*Fhb* spread.

Recent advances in genetic and genomic technologies have enabled the dissection of complex traits and find a novel resistant sources in wheat^37,47^. In order to provide novel resistant sources, we identified 15 unique QTLs linked to FCR resistance under GR and GH trials. Among them, eight QTLs on chromosomes 2AL, 3BS, 4BS, 5DS, 6DL were previously reported to be linked to FCR disease in wheat^9–13,19,48^. A QTL on chromosome 3BS has shown the strongest effect against *F. graminearum*^10^, however our study revealed only a moderate effect to *F. culmorum*. The genotyping platform and pathogen specific responses might explain the differences in expression of the QTL. However, several studies have shown 3BS region to be constantly linked to FHB resistance suggesting that this region may be highly conserved for major CR diseases (Fig. 6)^25,49,50^. Further, Collard *et al*.^9^ and Wallwork *et al*.^51^ identified a QTL on chromosome 4BS located close to dwarfing gene *Rht-B* in double haploid wheat populations which supports our finding^9,51^. Interestingly, we also detected 3 novel QTLs on chromosomes 3AS, 4BS and 5BS for both GR and GH conditions (Table 2). The detection of similar QTLs/loci in close genomic proximity suggests that these regions are highly conserved and are associated with FCR disease at multiple environmental conditions.

A large number of studies have identified numerous loci for FHB resistance in wheat. Despite this progress, genes/proteins underlying these QTLs have remained largely unexplored. Recently, wheat genomic resources have improved rapidly and a fully annotated reference genome (International Wheat Genome Sequencing Consortium (IWGSC), 2018) further extended the limitations. However, the lack of genomic and transcriptomic data still forms a substantial barrier e.g. in performing a molecular characterization of resistance/susceptibility genes. So far, most of these studies are linked to *F. graminea*rum in wheat^47,52–54^. The functional annotation of significant markers identified the majority of genes/proteins underlying QTLs to be associated with plant biological processes (Table 3). Most of the genes were linked to carbohydrate transport and metabolism, signal transduction, cell cycle control and response to stimuli (Table 4). Notably, the regions of the QTLs on chromosomes 4BS (*Fhb4*) and 6DS under GR, and QTLs on chromosomes 3BS (*Fhb1*), 4BS (*Fhb4*) and 6BS (*Fhb*2) under GH were previously reported to confer resistance to *F. gramenarium* disease incidence, spread and severity^17,25,26^. Further, the annotation of the flanking sequences of SNP marker linked to the QTL at 3BS identified NAD(P)(+)-binding protein linked to *Fhb1* (Table 3). High expression of NAD(P)(+)-binding proteins were reported with *Fhb1*(+) NIL line compared to *Fhb1*(-) NIL suggesting that NAD(P)(+)-binding proteins enhance FHB resistance^28^. NAD(P)(+)-binding proteins bind nicotinamide dinucleotide (NAD) to stimulate reactions central to energy production, storage, and transfer which are essential to nearly all core metabolic pathways in plants including photosynthesis. Further, glyceradehyde-3-phosphate dehydrogenase (GAPDH) is a NAD(P)(+)-binding protein and has been found to be involved in photosynthetic metabolism, abiotic and biotic stresses^55–57^. Furthr, Kugler *et al*.^58^ reported that jasmonate and ethylene biosynthesis genes were induced by *Fhb1*^58^. In general, *Fusarium* inoculation increased transcripts related to carbohydrate metabolism during early stage of parasitism including the starch and sucrose metabolism as well as glycolysis/gluconeogenesis pathways^47^.

CR resistance in wheat is a complex trait regulated by multiple loci^12^. This was comfirmed by our studies. We found that the majority of epistatic interactions involved minor QTLs, however, in one case an epistatic interaction between two major QTLs was recorded. Previous studies showing the epistatic interactions between the QTLs as well as QTL × environment were similar to our findings^59,60^. Many other studies have highlighted the role of environment in expression of the QTLs linked to FCR and FHB resistance^20,21,61^. Our study revealed that the main effect QTLs lead to increased resistance response under multiple environmental conditions (Tables 2 and 3). We identified two major QTLs on chromosomes 5AS and 7AL that were previously reported to harbor *Fhb1* resistance to FHB infection and severity^29^. Although these two QTLs as had been described earlier as major QTLs, our study detected them as only minor QTLs under GR. The use of a high density SNP genotyping platform and environment-depending responses might have separates QTLs as major or minor effects in our study. In addition to main/minor effect QTLs, there might be many other QTLs which were not significant at given *p-value* during QTL analysis but may have significant interaction with environments. Further work is required to validate these QTLs in FCR infection and development.

Our study indicated that FCR resistance in wheat is complex and controlled by multiple QTL/gene/s network and environmental effects. Further understanding of the interactions between the QTLs and with their environment may provide new insights into the mechanisms of FCR resistance. At the moment, we cannot predict if the identified QTLs carry only one or more resistance genes. Additional functional analyses will validate the actual role of these QTL/gene/s in host-parasitism. We conclude that the QTLs identified in this study are unique and valuable factors facilitating breeding resistance not only for FCR but also for other pathogens across multiple environments.

## Materials and Methods

### Plant materials and evaluation of resistance to FCR

A diverse set of 161 wheat accessions comprising of 101 breeding lines, 58 cultivars, and 2 landraces from IWWIP were tested for CR resistance. These accessions were selected based on genetic diversity and geographic origin. The details of the accessions including origin, accession number, and pedigree can be found in Table S1. The seeds were obtained by single-seed descent.

A highly aggressive monosporic isolate of *F. culmorum* was prepared on synthetic nutrient agar plate at 23 °C ± 1 °C under florescent lights with a 12 h/12 h day/night photoperiod for 10 days. A quarter of oven bags (35 cm × 48 cm) were filled with wheat bran. These bags were humidified, sealed with cotton and autoclaved at 121 °C for 20 min over three successive days. Spore suspension was prepared by adding sterilized distilled water to *F. culmorum* cultured synthetic nutrient agar plate, and added to the cool autoclaved wheat bran^19^. Inoculated wheat bran was mixed thoroughly and incubated at 23 °C for 3 weeks. After 3 weeks, fungus-colonized wheat bran was dried at room temperature and were used as inoculum for both GR and GH experiments.

For growth room trials, seeds were germinated on moistened tissue paper on Petri dishes for three days at 22 °C and transferred into RLC4-pine tubes (2.5 cm in diam. X 16 cm in depth, Ray Leach Cone-tainer^TM^, Stuewe & Sons, Inc. Oregon, USA) filled with a standard mixture of sand, soil, and organic manure (50:40:10; v/v/v). Soil mixture was sterilized at 121 °C for 2 h at 0.1 Mpa. The spore suspension was prepared by adding sterilized distilled water to fungus inoculated wheat bran and filtered through two layers of cheesecloth^19^. Each seven days old seedling stem base (0.5–1 cm above the soil level, including the coleoptile) was inoculated with 1 × 10^6^ spores ml^−1^ of *F. culmorum* spore suspension. Inoculated seedlings were covered 48 h with plastic sheet to maintain high humidity (80–90%). Plants were grown under GR at 23 ± 1 °C day/night temperatures with photoperiod of 16/8 h, and relative humidity of 60% (±5%) in a randomized complete block design (RCBD). Plants were watered from the bottom of the tray. Five plants of each accession were analyzed for CR resistance.

For greenhouse trials, two seeds of each wheat accession were grown in a SC10 super-pine tube (3.75 cm in diam. X 20.62 cm in depth, Ray Leach Cone-tainer^TM^, Stuewe & Sons, Inc. Oregon, USA) filled with sterilized soil mixture (same as GR). 0.5 g fungus-colonized wheat bran was used as inoculum source. The environmental condition under GH was controlled at 25/15 °C day/night temperatures, 16 h/8 h light/dark photoperiod, and relative humidity of 60–80% (±5%). Plants were watered at the crown base as needed. Plants were subjected to water stress at maturity stages to facilitate the disease development. The experiment was arranged in a RCBD with five replications. Experiments in both GR and GH were repeated twice.

Plants were harvested 42 and 49 days after transplanting under GH and GH, respectively. Disease severity was scored by visual inspection on the basis of browning intensity at the crown and the main stem (expressed in percentage). The following CR score was used: resistant (1: 1–9%), moderately resistant (2: 10–29%), moderately susceptible (3: 30–69%), susceptible (4: 70–89%), and highly susceptible (5: 90–99%)^19^. The widely grown winter wheat *cv*. Bezostaya 1 in Turkey was used as the susceptible control. The phenotypic data were analyzed using a mixed linear model implemented in Proc mixed procedure in SAS 9.4 (SAS Institute Inc., Cary, NC). Variance components were estimated according to the following model:

$${{\rm{Y}}}_{{\rm{ijk}}}={\rm{\mu }}+{{\rm{year}}}_{{\rm{i}}}+{{\rm{block}}}_{{\rm{k}}}({{\rm{year}}}_{{\rm{i}}})+{{\rm{accession}}}_{{\rm{j}}}+{{\rm{accession}}}_{{\rm{j}}}\,{\rm{by}}\,{{\rm{year}}}_{{\rm{i}}}+{\varepsilon }_{{\rm{ijk}}}$$

where “Y_ij_” is response variable; “μ” is overall mean; “year” is the random effect of year; “accessions_j_” is the fixed effect of the accession, “block_k_” is the random effect of the block with year, and “ε_ijk_” is the random error. The data were analyzed by restricted maximum likelihood to fit a mixed model. The total genetic variance (σ²_g_) and total environment variance (σ²e) were estimated by using PROC VARCOMP in SAS 9.4. In addition, broad sense heritability (H²) was calculated according to Holland *et al*.^62^. H² = σ²_g_/(σ²_g_ + σ²_e_/n), where ‘n’ is the number of environments. Further, Pearson correlations and regression between traits were calculated for both environments using Sigmaplot 8.

### DNA isolation and genotyping by 90K Illumina iSelect wheat bead chip

Genomic DNA was isolated from leaf tissue of seven days old wheat seedling using the Cetyl trimethyl ammonium bromide (CTAB) method^63^. The quality of DNA was evaluated on a 0.8% agarose gel and normalized to 50 ng/μl. A DNA aliquot of 2 μl from each sample was used for genotyping. Genotyping with 90K Illumina iSelect Wheat Bead Chip was performed at TraitGenetics GmbH, Gatersleben, Germany. To avoid monomorphic and low-quality SNPs, genotyped data were analyzed by Genome Studio software (V2011.1) and transcribed into binary matrix software^36^. All monomorphic markers, number of missing data greater than 5%, and SNP markers with minor allele frequency (MAF) less than 0.05 were culled to control false positive QTLs^64^.

### Linkage disequilibrium

In order to determine the pattern of linkage disequilibrium (LD) decay across 21 wheat chromosomes in three sub-genomes and whole genome, the LD squared correlation coefficients (*r*^2^) were calculated between marker pairs and plotted against the genetic distance in centiMorgans (cM) by SAS 9.4. Out of 23,364 polymorphic SNPs, the available markers with chromosomal positions were 20,116 SNPs and were used for the LD estimation (37, updated 2016). A cut off of (*r*^*2*^) = 0.1 was considered as the critical distance up to which a QTL region extends in this population^24^.

### Principle component and relatedness analysis

Principal component analysis (PCA) was performed with a total of 22,364 polymorphic SNP markers as covariance matrix which is further used for GWAS by TASSEL v.3.0 program^65^. Principle components (PCs) were estimated by Princomp procedure in SAS 9.4 to clarify the population structure and the significant level of PCs were evealuted by using the methods described by Franklin *et al*.^66^. Further, the relative kinship coefficients (K-matrix) among all pairs of accessions were calculated for GWAS analysis. PCs were treated as fixed effects and kinship (K matrix) was used to analyze the variance and covariance structure of random individual effects^39^.

### Genome wide association studies (GWAS)

GWAS was performed by adopting the multilocus mixed linear model (MMLM-P + K) that accounts for population Structure (P-matrix) and kinship (K-matrix)^38,67^ using the PROC MIXED in SAS version 9.4 (SAS Institute, Cary, NC). A total of 23,364 polymorphic SNPs markers, with <5% missing data and MAF < 5%, were used for QTL analysis. The same marker set was further used to calculate K-matrix (included as covariate for population stratification) and the P-matrix (PCA), to adjust population structure. The GWAS model used in this study described below: *y* = *Xβ* + *Pα* + *Iu* + *e*, where *y* is the vector of FCR score; *X* is the vector of the SNP markers, *β* is the vector of the allele effect to be estimated, P represent the first 3 PCs, while *α* represents the degree to which each PC explains SNP variation, *u* is the vector of the random effects for co-ancestry relations, and *e* is the vector of the residuals. To correct for the multiple testing, the threshold for accepting significant marker-trait associations was set at *p* = 0001, which was calculated as: 1/total number of SNPs^68,69^. Only those SNP loci that met the above criteria were examined and reported. Thereafter, the extent of LD decayed to *r*^2^ = 0.1 was used to defined the local LD-based QTL interval for each of the significant SNP^24^.

### Epistatic interaction

Genome-wide epistatic interactions were tested using the “interactions function” as implemented in the PROC MIXED procedure in SAS 9.4 by fitting a linear model with (P + K) variables, additive effects of the markers and their interactions. The *p*-value cutoff was set at 1 × 10^−6^ for accepting a significant gene-gene interaction effects. The statistical significance threshold was determined by cross validation and 1000 random permutations under the additive model with interaction. The interaction graph was drawn using the software Circos 0.69–6^70^.

### *In-silico* annotation of putative genes linked to FCR resistance

To identify and characterize the biological functions of putative genes, we performed an *in-silico* functional annotation of markers linked to FCR resistance. The flanking sequences of the significant markers were blasted against gene models of *Brachypodium distachyon, Oryza sativa*, and *Sorghum bicolor*, IWGSC, the Institute for Genomic Research (TIGR) Wheat genome annotation, and National Center for Biotechnology Information (NCBI). The genes/proteins were selected based on significant hit and lowest expect *e*-*value*^24^. The wheat transcriptome assemblies from MAS Wheat dataset^71^ were downloaded and the full open reading frame (ORF) at significant marker locations were identified. The data sets were imported into the CLC genomic workbench. The blast database sequences lower than *e-value* 0.0e-15 were considered positive. Further, if the significant marker was in coding region, the substitution was designated as synonymous (no change in amino acid) or non-synonymous substitution (change in amino acid).

## Supplementary information

Supplementary Table S1.
